# Impact on monthly migraine days of discontinuing anti-CGRP antibodies after one year of treatment – a real-life cohort study

**DOI:** 10.1177/03331024211014616

**Published:** 2021-05-17

**Authors:** Andreas R Gantenbein, Reto Agosti, Claudio Gobbi, Dominique Flügel, Christoph J Schankin, Dragana Viceic, Chiara Zecca, Heiko Pohl

**Affiliations:** 1 Department of Neurology and Neurorehabilitation, RehaClinic group, Bad Zurzach, Switzerland; 2 Department of Neurology, University Hospital Zurich, Zurich, Switzerland; 3 Kopfwehzentrum Hirslanden, Zurich, Switzerland; 4 Department of Neurology, Neurocenter of Southern Switzerland (NSI), Ospedale Regionale Lugano Civico, Lugano, Switzerland; 5 Faculty of Biomedical Sciences, Università della Svizzera Italiana (USI), Lugano, Switzerland; 6 Department of Neurology, Kantonsspital St. Gallen, St. Gallen, Switzerland; 7 Department of Neurology, Inselspital, Berne University Hospital, University of Berne, Berne, Switzerland; 8 Centre Médical Montchoisi, Swiss Medical Network, Lausanne, Switzerland

**Keywords:** Erenumab, galcanezumab, burden of disease, treatment interruption

## Abstract

**Objective:**

This study aims to analyse the effect of the discontinuation of anti-calcitonin gene-related peptide antibodies on monthly migraine days after 12 treatment months.

**Background:**

Anti-calcitonin gene-related peptide antibodies have been a game changer in migraine prophylaxis. However, high treatment costs warrant reducing treatment duration to the essential minimum.

**Methods:**

We collected data of patients with migraine who had received anti-calcitonin gene-related peptide antibodies and had received treatment for 12 months.

**Results:**

We included 52 patients. The average number of monthly migraine days was 16 ± 7 days at baseline, 6 ± 6 in the third, and 5 ± 4 in the 12th treatment month. After treatment interruption, the number of monthly migraine days was 6 ± 4 days in the first month, 9 ± 4 days in the second, and 11 ± 5 days in the third month. Most patients (88.9%) restarted treatment.

**Conclusion:**

Only little of the therapeutic effect of anti-calcitonin gene-related peptide antibodies outlasts their pharmacological effect. After treatment interruption, migraine frequency rose in most patients, and prophylaxis was required again in most cases.

Limiting treatment to benefitting patients and confirming the need for prophylaxis periodically is reasonable. However, our data does not support the need for prescheduled treatment discontinuation after 12 months and a fixed duration of the treatment interruption of 3 months.

## Abbreviations

CGRP = calcitonin gene-related peptide

MMD = monthly migraine days

## Introduction

Monoclonal antibodies targeted against calcitonin gene-related peptide (CGRP) or its receptors have been a game changer in migraine treatment. For the first time, a drug was developed explicitly for migraine prevention (1). Growing numbers of prescriptions document the success of these medications and hint at migraineurs’ unmet needs (2).

So far, available safety data have not raised concerns against long-term therapy (3–6). However, given the high treatment costs, a limitation of the treatment duration to the essential minimum is desirable. Besides, given the fluctuations of the attack frequency during migraineurs’ lives (7), eventually, prophylactic treatment might not be necessary any longer. Accordingly, guidelines generally suggest re-evaluating the need for migraine prophylaxis after 6–12 months (8,9).

Deciding about treatment interruption would be facilitated if the consequences were predictable. In particular, there are two urgent questions. First, how does treatment cessation influence the attack frequency? Second, is there a minimum period necessary to decide whether a patient requires treatment again? Swiss reimbursement rules have created a unique situation that allows answering these questions under real-life conditions.

First, only neurologists can prescribe anti-CGRP antibodies, with erenumab, galcanezumab, and fremanezumab currently being available (10). Second, treatment with at least two drugs licenced for migraine prophylaxis – that is, beta-blockers, calcium antagonists, or anticonvulsants (10) – must have been ineffective, contraindicated or not tolerated. Third, the mean number of monthly migraine days (MMD) in three consecutive months must be at least eight before the prescription can be made.

Patients taking erenumab are allowed to increase the dose after 3 months from 70 to 140 mg if the number of MMD did not drop by at least 50%. Treatment must be discontinued after 6 months if the MMD were not reduced by at least 50%. It must be halted in any case after 12 months and may be resumed 3 months later if the MMD rose to eight or more again (11). Exceptions from these rules are possible but need to be negotiated individually with insurance companies unless patients bear treatment costs themselves.

This study aimed to analyse the effects of the discontinuation of anti-CGRP antibodies on MMD after 12 successful treatment months.

## Methods

### Study design and data collection

Participating neurologists and headache experts from five study centres sent the fully anonymised data to one of the authors (HP) who conducted the statistical analysis. HP and CJS did not provide patient data. Data collection started in October 2020 and ended in December 2020. Inclusion criteria were a migraine diagnosis (episodic and chronic), and treatment with monthly injections of a monoclonal CGRP antibody for 12 months. We excluded patients if the provided data were obviously faulty. We included each centre’s first consecutive patients who met the inclusion criteria. The sample size was based on the available data.

We collected the following information: Age, sex, headache diagnoses, name of the prescribed antibody, MMD in the 3 months preceding the treatment (baseline), at 3 months and 12 months of treatment, and in the first, second, and third month after treatment cessation, as well as in the first month after treatment restart. Furthermore, we assessed adverse events and the reasons not to restart treatment after the interruption (if applicable). To limit the risk of a reporting bias, we asked neurologists to contribute data of their first patients treated with anti-CGRP antibodies.

No formal approval of an ethics committee was necessary, because neurologists provided strictly anonymised and routinely collected data of their patients and because the person undertaking the statistical analysis was unaware of their identity. Therefore, this study did not fall under the Human Research Act (12) and obtained a waiver from the concerned ethics committees (Req-2020-01324).

### Statistical analysis

We describe continuous variables as means, standard deviations, and ranges, proportions as percentages and categorical variables as frequencies. Missing values are referred to as not reported (nr).

Also, we determine the lowest possible number of migraine days in the first and second month after the interruption, predicting that a patient will have eight or more migraine days in the third month and therefore qualify for treatment with anti-CGRP antibodies again.

We used IBM SPSS Statistics version 25 for the calculations.

### Data availability

The data collected and analysed for the current study are available from the corresponding author on reasonable request.

## Results

We received data from 52 patients (47 females; 90.4%) with an average age of 48 ± 12 years (range 19–74 years). No data had to be excluded. Of these, 21 suffered from chronic migraine, 26 from episodic migraine without aura, two from episodic migraine with aura, and three from episodic migraine with and without aura. Besides, four (4/52, 7.7%) had received the diagnosis “medication overuse headache” at baseline. Patients took erenumab (51/52; 98.1%) – in the 12th treatment month, 24 received doses of 70 mg, and 27 of 140 mg – or galcanezumab (1/52, 1.9%).

Most patients experienced no adverse events (41/52, 78.8%); seven (7/52, 13.5%) reported constipation, two patients (2/52, 3.8%) complained about muscle cramps, one patient (1/52, 1.9%) about itching, one about flu-like symptoms, and one about an increase in headache days during the first 2 weeks after the first injection.

During the 3 months preceding the first injection (baseline), there were, on average, 16 ± 7 MMD (range 6–30). Furthermore, there were 6 ± 6 MMD (range 0–28; 1 nr) after three, and 5 ± 4 MMD (range 1–20; 1 nr) after 12 treatment months. Within the first year, the average reduction in MMD was 11 ± 6 days (range 2–29; 1 nr), and the average percentage reduction was 68.9% (SD 20.3, range 23.5–96.7%).

After 12 months, 45 patients (45/52, 86.5%) interrupted treatment with anti-CGRP antibodies. Of them, 40 (40/42, 88.9%, 3 nr) restarted treatment, averagely after 13 ± 3 weeks (range 8–20, 8 nr). The reasons not to restart were the patient’s subjective feeling of lacking efficacy and in another case, the patient’s impression that after having changed the workplace, a migraine prophylaxis might not be necessary anymore.

In eight patients, it was unknown when treatment was restarted; six patients already picked up treatment after 2 months (6/32, 18.8%, 8 nr). Consequently, in the first and second month after treatment interruption, 34 patients, and 28 in the third month had not restarted treatment yet. Among them, the number of MMD was 6 ± 4 days (range 0–15 days) in the first month, 9 ± 4 days (range 2–19 days) in the second month, and 12 ± 5 days (range 1–21 days) in the third month. Among those who had interrupted treatment for at least 3 months, the number of MMD was 6 ± 4 days (range 2–15 days) in the first month after treatment was started again.

Compared with the 12th treatment month, the MMD increased in 18 of 34 patients (52.9%) in the first month, and in 30 of 34 patients (88.2%) in the second month after treatment cessation. In the third month, six patients restarted treatment and MMD increased in 25 of the remaining 28 patients (89.3%) compared with the last month on treatment.

In the third month without treatment, MMD were as high as or higher than at baseline in seven patients (7/28; 25.0%); 24 of 28 patients (85.7%) had eight or more MMD and, thus, qualified for treatment with CGRP antagonists. Compared to baseline, MMD in the third month dropped by 25% (SD 49.7, range −163–97.0%) on average.

In the first month after the interruption, the lowest cut-off value predicting that a patient would have eight or more MMD in the third month, which resulted in zero false positives, was eight MMD; sensitivity was 0.190 because 17 of 21 were false negatives.

In the second month of the treatment interruption, the best cut-off value was 10, with zero false positives, and a sensitivity of 0.222 (14 of 18 were false negatives). Applying a cut-off value of eight would result in one false and 14 true positives (specificity 0.933), as well as 14 false and four true negatives (sensitivity 0.222).

Of all patients who had interrupted treatment for at least 3 months and had eight or more MMD in the third month, more than half had already had at least 8 days in the preceding months (14/24, 58%, 4 nr).

Table 1 summarises differences in MMD between patient with episodic and chronic migraine.

**Table 1.** Differences between patients with episodic an chronic migraine; MMD – monthly migraine days.

	Episodic migraine	Chronic migraine
MMD at baseline	14 ± 7 (n = 31)	20 ± 5 (n = 21)
MMD after 3 treatment months	6 ± 5 (n = 31)	7 ± 6 (n = 20)
MMD after 12 treatment months	5 ± 4 (n = 31)	5 ± 4 (n = 20)
MMD during the first month after treatment interruption	8 ± 4 (n = 19)	5 ± 3 (n = 15)
MMD during the second month after treatment interruption	9 ± 5 (n = 19)	8 ± 4 (n = 15)
MMD during the third month after treatment interruption	12 ± 6 (n = 14)	11 ± 4 (n = 14)
MMD during the first month after treatment restart	7 ± 5 (n = 8)	5 ± 3 (n = 9)

## Discussion

In this study, we analysed the MMD following discontinuation of anti-CGRP antibodies after 12 months of therapy. Treatment interruption resulted in an increase in MMD in almost all patients within 3 months. Half of them reached a migraine frequency that was as high as or higher than at baseline. Nevertheless, the MMD were, on average, still reduced by 25% in the third month after the last dose.

Reimbursement authorities insist on an interruption, probably attempting to reduce costs and avoid unnecessary treatment, especially since long-term data were initially lacking. However, our data show that all patients are at risk of relapsing into high numbers of MMD. Given the tremendous impact of frequent migraine attacks on migraineurs’ lives (13,14), it is a medical, social and economic imperative to prioritise preventing the migraine frequency from rising again. Hence, the interruption should be as short as possible.

According to Swiss governmental bodies, patients with at least eight or more migraine days in the third month after the last injection may resume treatment (11). Our data indicate that it is often unnecessary to wait 3 months for patients to meet these criteria. The ictal burden continues to increase after treatment cessation, and almost all patients who reach the threshold of eight migraine days in the first or the second month will have eight or more migraine days in the third month, too. Therefore, we suggest not postponing treatment restart when the number of MMD has reached the threshold value again.

Interestingly, despite differing baseline values, the number of MMD dropped to similar values in patients with episodic and chronic migraine (see Table 1), implying a greater reduction in MMD in chronic migraine. This finding is in line with previous studies suggesting that erenumab leads to a greater reduction in the number of migraine days in chronic migraine (3,15,16). After the interruption, MMD rose somewhat more slowly in chronic migraine. Consequently, it is possible that CGRP in the peripheral nervous systems is more relevant in the pathophysiology of chronic than episodic migraine.

An important question is whether treatment interruption after 12 months is justified in migraine patients at all. Since most patients in this study restarted the therapy, an interruption after 12 months could be too early. It is unlikely that the cost saved by the treatment discontinuation outweighs the increased disease burden the patients had to bear, especially given that the treatment was very well tolerated. We encourage studies investigating changes in the disease burden prospectively.

**Figure 1.**
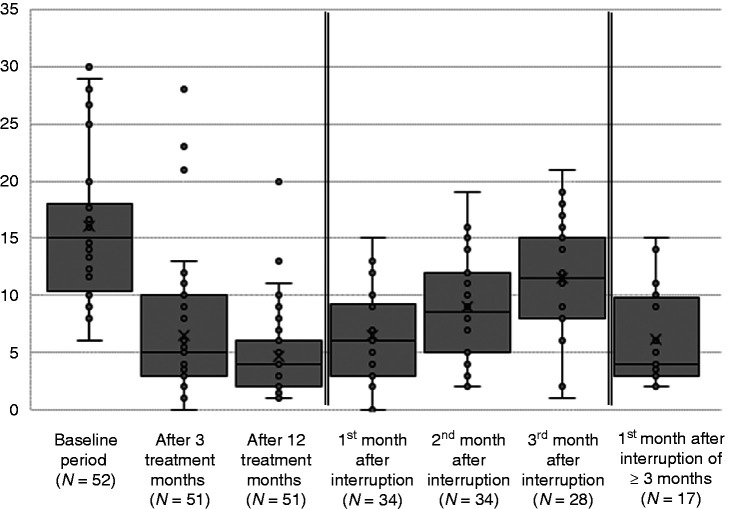
Monthly migraine days at different time points.

In accordance with a recent study (17), our data show that migraine frequency generally rises quickly after treatment interruption (see Figure 1). This finding suggests that most of the therapeutic effect does not outlast the pharmacological effect. However, about half of the patients did not reach their baseline number of MMD after 3 months. Given the half-life of about 28 days (18), plasma concentration has dropped by seven eighths in the third month after discontinuation. Consequently, it is unlikely that the lasting effect is due to the small proportion of the drug remaining in the body.

There may be several reasons for the persisting treatment effect. First, any medication overuse headache present at baseline probably would have resolved after 12 months in most 50% responders. Second, high numbers of MMD at baseline might have contributed to an increased attack frequency; for example, through central sensitisation. Third, lower attack frequencies during the treatment period might have relieved stress that many patients recall as a migraine trigger (19). Finally, even a disease modification effect could be speculated.

## Strengths and limitations

Because of the requirements for the reimbursement of anti-CGRP antibodies (11), we can assume that migraine diagnosis was correct, and there was accurate documentation of MMD in the diaries. Besides, after 12 months, plasma concentration had reached a steady state. Hence, all patients had halted treatment under similar conditions and study results were generalisable.

A limitation is that we had asked participating neurologists to report their first patients treated with anti-CGRP antibodies to prevent a reporting bias. These patients might have been the first to receive the treatment because they were exceptionally severely affected. Hence, we cannot completely rule out a sampling bias. Confounding factors, such as concomitant preventive medication, have not been fully reported but are expected to be in a very low range. Nevertheless, we cannot exclude that additional migraine prophylaxis had an influence on the data.

In addition, the collected data does not allow verifying that all patients had been treatment responders after six treatment months. Nevertheless, we assume that all participating neurologists would have interrupted the treatment had patients been non-responders.

Finally, the sample size was rather small; hence, generalisability may be limited.

## Conclusion

Only a small proportion of the therapeutic effect of anti-CGRP antibodies outlasts their pharmacological effect. In our sample, migraine frequency rose in most patients after treatment interruption, and usually, prophylaxis was required again soon.

It is reasonable to limit prophylactic treatment to those who benefit and to confirm the need for prophylaxis periodically. However, our data do not support the need for prescheduled treatment discontinuation after 12 months and a fixed duration of the treatment interruption of 3 months.

## Clinical implications

- After the interruption of the treatment with antibodies directed against CGRP or its receptors, migraine frequency rises quickly in most patients, and prophylaxis was usually required again soon.
- In the third month after treatment interruption, migraine frequency had not reached baseline values in most participants, suggesting that a small portion of the therapeutic effect of anti-CGRP antibodies outlasts their pharmacological effect.
